# Mechanisms of Aminoglycoside Ototoxicity and Targets of Hair Cell Protection

**DOI:** 10.1155/2011/937861

**Published:** 2011-10-25

**Authors:** M. E. Huth, A. J. Ricci, A. G. Cheng

**Affiliations:** ^1^Department of Otolaryngology-Head and Neck Surgery, Stanford University School of Medicine, 801 Welch Road, Stanford, CA 94305-5739, USA; ^2^Department of Otorhinolaryngology, Head and Neck Surgery, Inselspital, University of Bern, Freiburgstrasse, 3010 Bern, Switzerland; ^3^Department of Molecular and Cellular Physiology, Stanford University School of Medicine, 300 Pasteur Drive, Stanford, CA 94305, USA

## Abstract

Aminoglycosides are commonly prescribed antibiotics with deleterious side effects to the inner ear. Due to their popular application as a result of their potent antimicrobial activities, many efforts have been undertaken to prevent aminoglycoside ototoxicity. Over the years, understanding of the antimicrobial as well as ototoxic mechanisms of aminoglycosides has increased. These mechanisms are reviewed in regard to established and potential future targets of hair cell protection.

## 1. Introduction

Aminoglycosides (AGs) are a well-known and successful class of antibiotics. The initial isolation of streptomycin from *Streptomyces griseus* provided the long-sought treatment for tuberculosis and an effective antibiotic against gram-negative bacteria [1, 2]. In subsequent years, other AGs were isolated from *Streptomyces* spp., commonly integrating the ending “-mycin” in their nomenclature [3, 4]. With the isolation of gentamicin from Micromonospora purpurea [5], the ending “-micin” was implemented to specify the bacterial origin of the individual AG. In contrast to these organic derivatives of soil-dwelling bacteria, synthetic AGs such as amikacin could be developed *in vitro* [6]. Currently, nine AGs (streptomycin, neomycin, tobramycin, kanamycin, paromomycin, spectinomycin, gentamicin, netilmicin, and amikacin) are approved by the Food and Drug Administration (FDA) [7]. 

In addition to their potent antimicrobial efficacy, all AGs can cause toxic side effects to the kidneys and inner ear. While damage inflicted by AG on the kidney is usually reversible [8, 9], damage to the inner ear is permanent [10]. This nephro- and ototoxicity was initially discovered in the first clinical trials of streptomycin [11, 12]. Within the inner ear, streptomycin preferably damages the vestibular organ [12]. Modification of streptomycin to dihydrostreptomycin, however, resulted in a shift of ototoxic damage from the vestibular organ to the cochlea [13]. Generally, each AG is capable of irreversibly damaging both the auditory and vestibular organs, but “typically affects one more than the other” [14]. Gentamicin and tobramycin are predominantly vestibulotoxic, whereas neomycin, kanamycin, and amikacin are mainly cochleotoxic [15]. Ototoxic side effects occur within days or weeks after systemic application and are often bilateral in presentation [16]. Vestibulotoxicity occurs in up to 15% of patients after AG administration [17], whereas cochleotoxicity in 2% to 25% of patients [17, 18]. Different regimens of AG administration and different definitions of ototoxic damage may have contributed to the variation of incidence [19].

Symptoms of cochleotoxicity include hearing loss and/or tinnitus, while those of vestibulotoxicity consist of disequilibrium and dizziness. Unfortunately, these symptoms may not be detected until after the acute phase of severe infection and diagnosis is thus delayed. AG cochleotoxicity typically affects first the high frequency and then extends towards the lower frequency and ranges over time in a dose-dependent manner [20, 21]. Because the ultrahigh frequencies of hearing are not routinely tested (>8 kHz), the true incidence of AG-induced hearing loss is often underestimated. Indeed, when ultra-high frequency testing was performed, hearing loss was reported in 47% patients with a history of AG treatment [22]. 

Despite the nephro- and ototoxic side effects, AGs are still the most commonly prescribed antibiotics [23, 24]. In the industrialized world, use of AGs is usually limited to severe infections including those caused by multidrug resistant tuberculosis [25, 26]. Neonates frequently receive AGs for suspected or proven gram-negative infection, as sepsis is associated with high mortality [27]. In the developing world, however, AG use has been popular because of their low cost and potent antibacterial activities, outrivaling more expensive antibiotics with less severe side effects. There, AGs are even prescribed as first-line therapy for less severe conditions such as bronchitis or otitis media [28]. Additional safety precautions such as blood level monitoring or hearing tests are also limited [19]. As a result, the incidence of AG ototoxicity in developing countries may increase in comparison to the industrialized world.

## 2. Pharmacokinetics and Antimicrobial Mechanism of Aminoglycosides

The AG class of compounds consists of an aminocyclitol moiety with two or more amino sugar rings [29]. A characteristic quaternary ammonium group makes AGs polycationic (positive charge) and highly polar [30, 31]. As a result, enteral absorption is poor and AGs are generally administered parenterally or topically [32]. After parenteral administration, AG plasma levels peak between 30 and 90 minutes [7, 33]. Drug metabolism is minimal as approximately 99% of the administered AGs are eliminated unaltered by glomerular filtration in the proximal tubule [34, 35]. The plasma half-life of AGs ranges from 1.5 to 3.5 hours [7, 36], but is prolonged in neonates, infants, and conditions with decreased kidney function [7, 37].

The most common indication to administer AGs is for empirical treatment of patients with severe infections such as septicemia, nosocomial respiratory tract infection, complicated urinary tract infections, and complicated intra-abdominal infection [25], partly because AGs are shown effective against aerobic, gram-negative bacteria [38]. AGs demonstrate an increased selective antimicrobial activity in an alkaline environment [39]. It has been suggested that an alkaline pH compromises the bacterial membrane [40, 41], which might facilitate AG penetration into bacteria. Additionally, AGs have up to 6 amines with pKs varying more than two orders of magnitude, thus making the molecules much less charged at alkaline pH and so more able to interact with a lipid environment. Normally, the positively charged nature of the AG molecule precludes free passage through lipid barriers such as cell membranes, but promotes bacterial uptake and rapid binding to negatively charged lipopolysaccharides (LPS) in the outer membrane of gram-negative bacteria [42]. By competitive displacing bridging divalent cations such as Mg^2+^ or Ca^2+^, AGs can disrupt cross-links between adjacent LPS [43]. Such a disruption damages membrane integrity and leads to blebbing of the outer membrane, ultimately resulting in transient holes of the gram-negative cell wall [44, 45]. This formation of holes in the cell wall facilitates further AG uptake and appears to significantly contribute to the bactericidal effect of AGs [46]. With this first step, AGs enter the periplasmic space of gram-negative bacteria in a passive and non-energy-dependent manner [47]. In a second step (also referred to as energy-dependent phase I), AGs are transported further through the inner bacterial membrane in an oxygen-requiring process [47]. Therefore, uptake is facilitated in aerobic bacteria [47]. Once in the cytosol, AGs interact with the 30S subunit of bacterial ribosomes [48, 49] in a third and energy-dependent step (energy-dependent phase II) [47, 50]. At the 30S subunit, AGs bind to the decoding site located at the A site of the 16S rRNA [51, 52]. Binding of AGs at this site perturbs the recognition and selection of tRNA during translation and increases misreading [52, 53]. Furthermore, binding of AGs inhibits ribosomal translocation [54–56]. Perturbation of both ribosomal translation and translocation ultimately inhibits protein synthesis. Interestingly, the affinity for different rRNA binding sites varies amongst different classes of AGs [57–59]. This slightly different AG-ribosome interaction, therefore, appears to be beneficial against bacterial resistance [19].

## 3. Ototoxicity and Mechanism of Hair Cell Damage

### 3.1. Susceptibility and Genetic Predisposition for Aminoglycoside Ototoxicity

While AGs preferentially target the bacterial ribosome, the inner ear and kidney are known to receive collateral damage in many patients receiving treatment [11, 12]. However, a meta-analysis comparing once versus multiple-daily regimens of different AGs could not determine a statistical significant correlation between ototoxicity and treatment regimens [60]. One main susceptibility factor (17%–33% of patients with reported ototoxic damage [61]) is the genetic predisposition to AG ototoxicity [62]. The fact that this increased susceptibility was inherited maternally suggested mitochondrial involvement [62]. This is compelling in light of the endosymbiotic theory as mitochondrial ribosomes demonstrate more similarities to prokaryotic ribosomes than cytosolic ribosomes [63, 64]. Therefore, the small subunit of the mitochondrial ribosome is one of the primary targeting sites for AGs [48, 49]. 

Several mutations in mitochondrial DNA are linked to increased susceptibility to AG ototoxicity [61, 65, 66]. Exposure to AG leads to impairment of RNA translation within mitochondria through interaction with binding sites on mitochondrial 12S rRNA [65]. This interaction was mapped to an adenine-to-guanine mutation at nucleotide 1555 in the 12S rRNA gene [65]. Of additional note, bacterial resistance mutations are described at this locus [67, 68]. This mutation increases structural similarity of mitochondrial rRNA to bacterial rRNA [65], which promotes binding of AG to mutated mitochondrial 12S rRNA [69, 70]. As a result, damage can result from decreased protein synthesis [69]. Although no direct evidence exists to link ototoxicity to an inhibition of mitochondrial protein synthesis, inhibition of mitochondrial protein synthesis potentiates AG toxicity [71]. Also, electron microscopy reveals mitochondrial disruption following AG treatment [72].

This susceptibility mutation has been reported in 17%–33% of patient with reported AG ototoxicity [61]; in the general population of the European Union, it is estimated to be 1 : 500 [73, 74]. Other mutations leading to increased AG susceptibility have also been described, including C1494T [66]. The C1494T mutations have varying degrees of penetrance [75], are less common than the A1555G mutation [76], and are sporadic with multiple origins [77]. In sum, the prevalence of the most common mutations across varying ethnic backgrounds is 0.9%–1.8% [76, 78], of which 5%-6% are sporadic [63, 79, 80].

Although this genetic susceptibility is present in all organs, the mitochondrial mutations target the cochlea but not the vestibular organs or the kidneys [81]. This is intriguing as this selective cochleotoxicity also occurs with preferably vestibulotoxic AGs such as streptomycin [81]. One proposed explanation for this phenomenon is that AGs cause misreading in mitochondrial protein synthesis rather than direct inhibition of protein synthesis [82] such that tissues rich in mitochondria would be predominately affected [81]. Exposure to AGs would decrease mitochondrial ATP synthesis resulting in compromised ion pump activity [81, 82]. Reduced ion pump activity in strial intermediate cells could ultimately lead to a progressive decrease of the endocochlear potential [81]. This scenario conceivably explains the slow progression of hearing loss after exposure to AGs observed in patients with increased genetic susceptibility [81]. The strial impairment, furthermore, would explain the little effect on vestibular function in these patients [81]. Interestingly, the stria vascularis demonstrates extensive degeneration in syndromal mitochondrial diseases [83]. This further supports the hypothesis of the stria vascularis as the cochlear cells targeted by the mitochondrial mutations in patients with increased genetic susceptibility to AG ototoxicity. An alternative simple explanation is that susceptibility to the mitochondrial disease is a function of metabolic demand so that hair cells operating at higher frequencies will be more susceptible to a reduced mitochondrial function than lower frequency cells, that is, cochlea versus vestibular, basal versus apical, and type I versus type II. Similarly the highly metabolically active strial cells would also have increased sensitivity.

In genetically susceptible individuals, it is postulated that a single injection of AG can cause ototoxic damage [84], implying that genetic factors can reduce the threshold concentration at which AGs cause damage [61]. At higher concentrations or more frequent doses of AG, the incidence of ototoxic damage exceeds the prevalence of genetic predispositions [76, 81, 85]. Although *in vitro*, a clear relationship between damage and AG concentration is observed, the extent of ototoxic damage *in vivo* does not seem to correlate with AG concentration in targeted tissues [86]. This discrepancy requires further evaluation.

### 3.2. Route of Aminoglycosides into Hair Cells

**Figure 1:**
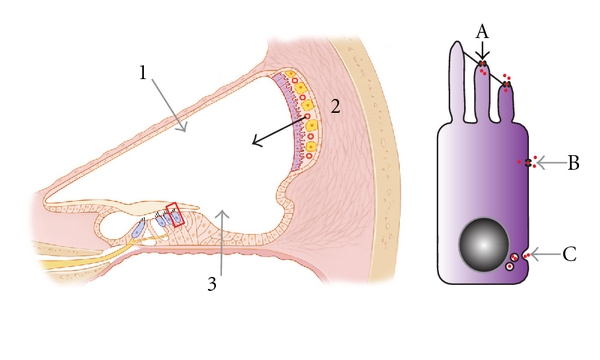
Proposed mechanisms of aminoglycoside transport in the inner ear. Possible entry sites for aminoglycosides into the scala media include via (1) the Reissner's membrane, (2) stria vascularis, and (3) basilar membrane. Published work supports the notion of entry via the Reissner's membrane and the stria vascularis through and between the marginal cells. At the hair cell level, aminoglycosides can potentially enter via mechanotransducer channels located on stereocilia of hair cells (A), endocytosis on the apical or basolateral membranes (A, B, or C), TRP channels (A, B or C), or ATP receptors (A).

After systemic administration, AGs are detected in the cochlea within minutes. Fluorescently labeled gentamicin was detected in the stria vascularis 10 minutes after injection in mouse [87]. In the stria vascularis, the fluorescently tagged gentamicin increased over time mainly in marginal cells, but also in intermediate and basal cells as well as fibrocytes, plateauing after 3 hours [87]. These observations suggest that gentamicin enters the inner ear fluids from the strial capillaries through the strial marginal cells [87]. In the organ of Corti, fluorescence from labeled gentamicin starts increasing 1 hour after systemic injection. Hair cells demonstrate fluorescent gentamicin intracellularly after 3 hours [87]. Earlier studies demonstrated similar pharmacokinetics in rat and guinea pig [88, 89]. In rat cochlear tissues, gentamicin concentrations were measured by a radioimmunoassay and peaked 3 hours after systemic application [89]. In guinea pig, gentamicin appeared in the stria vascularis 30 minutes after systemic injection. In outer hair cells (OHCs), gentamicin was detected after 30 minutes and peaked 6 hours after systemic injection [88]. Although these studies had different specific time points for measurements, they are roughly in agreement as to the time course of uptake into cochlear tissues [87–89]. Based on the cochlear structures, where AGs are located, entry into various cochlear structures suggests a complex uptake mechanism (Figure 1). 

Both endocytosis and transport through ion channels are proposed to mediate AGs uptake into sensory hair cells. While some publications describe endocytosis as the mechanism of entry into hair cells [90, 91] others advocate for the mechanoelectrical transducer (MET) channel located at the top of hair cell stereocilia [92–94]. The endocytic mechanism of AG entry arose because researchers observed the appearance of vesicles in the subcuticular region of hair cells after systemic injection in guinea pigs [95]. Hashino and Shero observed kanamycin in intracellular vesicles 27 hours after systemic injection in chicken [90]. These findings were interpreted as evidence for endocytosis as mechanism of AG uptake as the vesicle membranes contained cationic ferritin, a membrane bound marker [90]. However, no differences in intravesicular AG, compared to a control group, were observed until 12 hours after injection [90]. 

Myosin7a was hypothesized to play a role in endocytosis-mediated AG uptake due to its concentrated expression at the apical part of hair cells in a region with high amounts of vesicles known as the pericuticular necklace [91, 96]. The lack of AG uptake in Myosin7a^6j^ mutant mice was considered evidence supporting AG toxicity mediated by endocytosis [91]. Further investigation found that Myosin7a-deficient hair cells exhibit closed MET channels at rest, confounding initial interpretations [97].

Furthermore, the rate of endocytosis correlates with temperature and, therefore, is decreased in hypothermic conditions [98]. AG uptake demonstrates little temperature-dependent kinetics, indicating a minor relevance of endocytosis in the process [99]. Instead, there is strong evidence that AGs enter hair cells through the MET channel located at the top of the stereocilia. AGs act as open channel blockers of the MET-channel [100, 101]. Initially, AGs were not considered to be permeable because diameter estimates of the MET-channel pore were low (0.6 nm) [102]. However, work by Gale and coworkers suggested that larger molecules could pass through the MET channel [103]. This was quantified by Farris et al. with a new pore size estimate of 1.25 nm [104], which is large enough to pass AGs. Marcotti et al. demonstrated directly that AGs could pass through the channel [92]. Interestingly, this block was decreased significantly for AG approaching the channel from the internal as opposed to the external face [92]. As this difference between internal and external blocking of AG makes the MET channel function like a one-way valve, intracellular accumulation of AGs is promoted and might explain the increased susceptibility of hair cells compared to other cell types [94]. The significance of the MET channel as a major route of AG entry is furthermore supported by the exacerbation of ototoxic damage with noise exposure [105]. Acoustic stimuli increase the open probability of the MET channel and thereby, increase AG uptake [106]. Additionally, the distribution of ototoxic damage with increasing hair cell susceptibility from apex to base corresponds to the transduction currents in the cochlea, which are larger in basal than in apical OHCs and, in general, more decreased in inner hair cells (IHC) [107–109]. Moreover, fluorescently labeled gentamicin has been observed first in the tips of hair cell stereocilia before the fluorescent signal increases in the hair cell body [87].

Several other ion channels might also contribute to AG uptake into hair cells. Channels of the transient receptor potential (TRP) class such as TRPC3, TRPV4, TRPA1, and TRPML3 are expressed in the cochlea [110–112] and are permissive to AGs in kidney cells [113, 114]. It is unclear at this point under what conditions the TRP channels might be open and whether these channels are expressed in the plasma membrane or in other cytosolic compartments. The glycoprotein megalin is another potential mediator for AG uptake. Megalin is predominantly expressed in the proximal tubules of the kidney. Megalin is capable of binding AGs and is also expressed in the inner ear [115]. Therefore, it was considered a candidate protein for the uptake of AG into hair cells. However, megalin is a drug receptor engaging in endocytosis and is not expressed in the organ of Corti and sensory hair cells [115–117]. Notwithstanding, megalin has been detected in marginal cells of the stria vascularis, suggesting a role in the transport of AGs into the inner ear fluids [116, 117].

### 3.3. Apoptotic Pathways of Ototoxic Hair Cell Death

Inside the hair cell, AGs cause damage, either directly or indirectly, by first inducing disarray of stereocilia and ultimately ending with apoptotic cell death [118–121]. The presence of AGs within hair cells leads to increased formation of reactive oxygen species (ROS) or free radicals [122–125]. A common mechanism for the formation of ROS is the Fenton reaction:

(1) $${\text{Fe}}^{2+}+{\text{H}}_{2}{\text{O}}_{2}\to {\text{Fe}}^{3+}+{\text{HO}}^{∙}+{\text{HO}}^{-}.$$

Here, the presence of iron salts is required [126]. When gentamicin combines with iron salts, the gentamicin-iron complex enhances iron-catalyzed oxidations and, thereby, directly promotes the formation of ROS [122]. This requires electrons for which unsaturated fatty acids can act as electron donors. In return, those fatty acids, predominantly arachidonic acid, are oxidized to lipid peroxides [125, 127]. As arachidonic acid is an essential fatty acid present in cellular membranes, ROS can affect membrane fluidity and permeability [128, 129]. Via lipid peroxidation, ROS can also affect proteins and nucleic acids thereby disrupting the activity of enzymes, ion channels, and receptors [128–131]. ROS naturally occur in the cell as a regular byproduct of cellular metabolism [130–132]. Normally, the cell protects itself from lethal ROS accumulation with intrinsic antioxidants such as glutathione [132, 133]. This intrinsic protective system is capable of neutralizing ROS to some extent [134]. When formation of ROS, however, overwhelms the capacity of these intrinsic protective and repair systems, the cell then undergoes apoptotic cell death [135, 136].

The mechanism of involvement of mitochondrial mutations in ototoxic hair cell death is not completely understood. Exposure to AG leads to impairment of RNA translation and inhibition of protein synthesis within mitochondria [65, 69, 137]. It is further suggested that inhibition of mitochondrial protein synthesis leads to a decrease in ATP [137]. With the decrease of energy production, the mitochondrial integrity is compromised and predispose to a leakage of cytochrome c and subsequent activation of the apoptotic cascades. Furthermore, it is hypothesized that the mitochondrial RNA mutations when exposed to AG cause an increased formation of ROS, which then promote apoptotic cell death [137].

**Figure 2:**
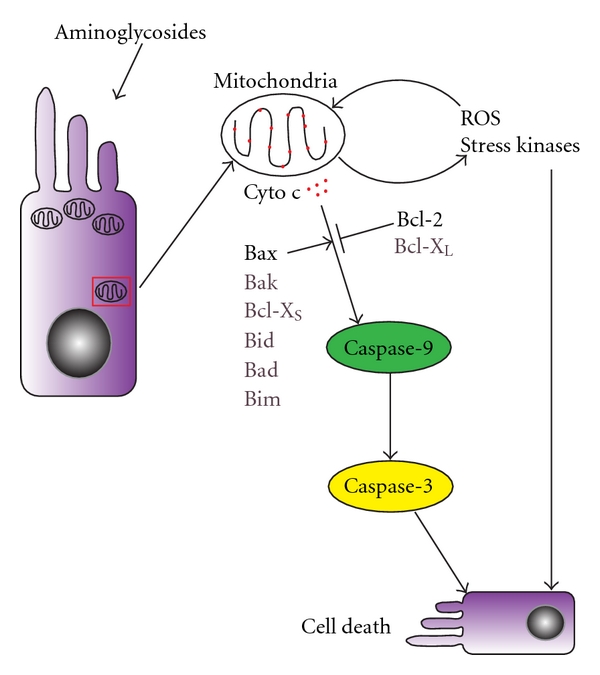
A simplified schematic of the cell death cascade in hair cells damaged by aminoglycosides. Reactive oxygen species (ROS), stress kinases, and the caspase family of proteases are activated and mediate hair cell degeneration caused by aminoglycoside exposure, whereas overexpression of Bcl-2 protects against caspase activation and hair cell loss. Aminoglycosides damage the mitochondria and can result in generation of ROS and activation of stress kinases. Both ROS and stress kinases can cause cell death directly as well as amplify insults targeting the mitochondria. The balance between pro-apoptotic and anti-apoptotic Bcl-2 family members determines the integrity of the mitochondria. Cytochrome c leaking out of damaged mitochondria leads to caspase-9 activation, which in turn activates caspase-3 to execute cell death.

Independent extrinsic and intrinsic apoptotic pathways exist [138, 139]. The extrinsic pathway is mediated by death receptors including the tumor necrosis factor (TNF) family. When stimulated, death receptors activate cysteine-dependent, aspartate-specific proteases also known as caspases. The prototype of death receptors is the FAS (CD95/APO-1) receptor, which activates caspase-8 on stimulation. Caspase-8 in turn initiates a cascade involving the activation of caspase-3, caspase-6, and caspase-7, which ultimately execute cellular degeneration [140]. The intrinsic pathway, in contrast, is the major apoptotic pathway initiated by aminoglycoside ototoxicity (Figure 2) [120]. The intrinsic pathway is predominantly triggered by nonreceptor stimuli such as cytokine deprivation, DNA damage, and cytotoxic stress [141]. Characteristic for the intrinsic apoptotic pathway is the permeabilization of the outer mitochondrial membrane resulting in leakage of proapoptotic factors from the mitochondrial intermembrane space into the cytoplasm. Mitochondrial membrane integrity and components of the intrinsic pathway are regulated by proteins of the B-Cell Lymphoma-2 (Bcl-2) family [141]. 

Bcl-2 is the prototype of this equally named protein family. Studies in other systems report these molecules as key apoptosis mediators, acting upstream of caspase activation [142–144]. The Bcl-2 proteins function as a checkpoint for cell death and survival signals in the mitochondria (Figure 2). The Bcl-2 protein family can be anti- or pro-apoptotic [136, 145, 146]; anti-apoptotic Bcl-2 proteins include Bcl-2 and Bcl-X_L_ [143, 147], whereas pro-apoptotic Bcl-2 proteins which promote cell death include Bax, Bak, Bcl-X_s_, Bid, Bad, and Bim [143, 147]. Bcl-2 proteins form hetero- and homodimers within the cell. When a cell is challenged, the balance between anti- and pro-apoptotic Bcl-2 proteins regulate whether or not apoptotic cell death is initiated [148]. Anti-apoptotic Bcl-2 proteins are able to bind to pro-apoptotic Bcl-2 proteins, thus neutralizing the pro-apoptotic signal [149]. When the balance moves in favor of apoptosis, the pro-apoptotic cytoplasmic Bcl-2 member Bax translocates to the mitochondria, causing pores in the mitochondrial membrane [143, 144]. This leads to loss of mitochondrial transmembrane potential, generation of ROS, and leakage of cytochrome c into the cytoplasm [143, 144, 150–154], thus activating the upstream caspase pathway as mentioned above. Supporting a role of this pathway in the inner ear, hair cell loss, and caspase-9 activation were prevented in utricles from Bcl-2 overexpressing mice when treated with neomycin [155]. This suggests a role for Bcl-2 in the upstream caspase cascade in aminoglycoside-induced hair cell death. 

Another group of mediators of apoptotic hair cell death is the stress-activated protein kinases, including the mitogen-activated protein (MAP) kinases (Figure 2) [120]. A particular group of MAP kinases are c-jun N-terminal kinases (JNK). These JNKs are located in the cytoplasm and regulated by c-Jun-interacting protein-1 (JIP-1) [156, 157]. In response to cellular insults, JIP-1 facilitates the phosphorylation and thus activation of JNK [158–161]. Activated JNK in turn phosphorylates and thereby activates the transcription factors c-Jun, c-Fos, ELK-1, and activated transcription factor 2 (ATF-2) in the nucleus and Bcl-2 in mitochondria [120]. After AG treatment, increases in JNK, c-Jun, c-FOS, and Bcl-2 have been reported in hair cells [120, 152, 161, 162]. Activation of the JNK signaling pathway appears to precede the release of mitochondrial cytochrome c, which then activates caspases [152, 163].

Caspases execute cell death in apoptosis [141]. The caspase family consists of 14 members in mammals, with only a subset involved in apoptosis [142, 164]. Caspases can thus be segregated into upstream and downstream enzymes, which are normally inactive [136, 164]. Caspases exist in the cytoplasm normally inactivated by inhibitor of apoptosis proteins (IAP) [136, 141]. Activation of upstream caspases occurs by apoptosis-inducing signals such as p53, which has been shown to activate caspases after administration of cisplatin. Downstream caspases are activated by upstream caspases through cleavage of an inactivating prodomain to produce the mature enzyme [136].

Caspase-8 is an upstream member that is tightly linked to membrane-associated death domain-containing receptors. When ligands such as Fas ligand or tumor necrosis factor alpha bind to this receptor, caspase-8 is recruited intracellularly, leading to clustering and autoactivation of other caspase-8 molecules [165]. This subsequently causes activation of downstream caspases such as caspases-3, -6, and -7. Although caspase-8 is detected in HC after AG administration [166, 167], it does not play a key role in HC death, as inhibition of this pathway does not prevent HC death or prevent caspase-3 activation [166, 168, 169]. 

Caspase-9 is an upstream caspase activated by apoptotic signals from the mitochondria. This pathway is initiated by cytochrome c release from mitochondria, which then binds to apoptosis protease activating factor, dATP, in the cytoplasm and procaspase-9 [164, 170]. This binding causes cleavage and activation of caspase-9, which subsequently cleaves and activates downstream caspases, ultimately resulting in apoptotic cell death (Figure 2). Activated caspase-9 is detected in cochlear and utricular hair cells after AG treatment *in vitro* [151, 166, 167]. 

Caspase-3 is a primary downstream caspase that executes the apoptotic program by cleavage of proteins necessary for cell survival, including Bcl-2, inhibitors of deoxyribonucleases, and cytoskeletal proteins (Figure 2) [171–174]. This enzyme activation has been detected in HC due to ROS after AG dosing [150, 151, 166, 167, 175]. 

Further mechanisms of apoptotic hair cell death following AG administration involve activation of NF-*κβ* as well as calcium-dependent proteases such as calpains. Inhibition of NF-*κβ* in rat cochlear explants after exposure to gentamicin altered the ratio of activated over inactivated pro-apoptotic factors such as c-Jun and p38 as well as of anti-apoptotic factors such as akt [176]. Exposure of mice cochlear cultures to neomycin resulted in apoptotic DNA fragmentation, which could be prevented by a calpain inhibitor [177].

Overall, apoptotic death of hair cells due to AG exposure is complex and our understanding of it has increased in recent years. A simplified model of the apoptotic cascade in aminoglycoside damaged hair cells is presented in Figure 2, but it is important to point out that many components of the overall cascade and the interactions among these components are still poorly understood. This complexity is in part reflected by crosstalks among pathways. Death receptor stimulation, for example, is also capable of activating the intrinsic pathway despite primary involvement in the extrinsic pathway [141].

## 4. Efforts in Hair Cell Protection

With increasing understanding of ototoxic cell death, a myriad of therapeutic efforts have been proposed to target various steps of the complex cascades to hair cell death. Those strategies include inhibition of apoptosis, neutralization of ROS, and administration of neurotrophic factors. A detailed overview of relevant studies including applied drugs, dosage, and outcome is presented in a table at the end of each subchapter.

### 4.1. Inhibition of Apoptotic Enzymes

Permeable caspase inhibitors such as z-Val-Ala-Asp(*O*-Me)-CH_2_F-fluoromethyl ketone (zVAD) were applied against different AGs in a variety of species. zVAD inhibits by irreversibly binding to the active site of a broad spectrum of caspases [167]. Caspase inhibitors conferred significant protection against hair cell damage from AG, preserving hair cell morphology as well as function *in vitro* and *in vivo* [167, 178–182] (Table 1).

Agents targeting upstream stress kinases in the apoptotic cascades also prevented AG-induced hair cell death. D-JNKI-1 is a cell permeable peptide that binds to all three isoforms of JNK, thereby blocking JNK-mediated activation of the apoptotic transcription factor c-Jun [156]. Inhibition of the MAP-JNK pathway by application of D-JNKI-1 prior to treatment with neomycin resulted in significant protection from hair cell loss *in vitro* and hearing loss *in vivo* [161]. Other JNK inhibitors that successfully prevented AG ototoxicity are CEP-1347, CEP 11004, and 17*β*-Estradiol [152, 183–186] (Table 1).

Targeting the Bcl-2 family as the upstream key mediator of apoptosis also prevented AG-induced hair cell loss. Overexpression of the anti-apoptotic Bcl-2 in transgenic mice significantly decreased hair cell loss and preserved hearing function following AG exposure *in vitro* and *in vivo* [155, 187]. Inoculation of mouse cochlea with an adenovirus vector expressing the anti-apoptotic Bcl-X_L_ before treatment with kanamycin also protected from hair cell loss and preserved hearing function [188] (Table 1).

Another class of stress-activated proteins are the family of heat shock proteins (HSPs), which are upregulated in stressed cells in multiple organ systems. HSPs can not only prevent protein aggregation by promoting proper folding of nascent or denaturated polypeptides [189], but also inhibit apoptosis. Induction of HSP expression in cultured mouse utricle led to upregulation of HSP-70, HSP-90, and HSP-27 [190]. Overexpression of HSP-70 in transgenic mice significantly protected from hair cell loss from neomycin treatment *in vitro,* but also significantly protected from hearing loss and hair cell death in mice injected with kanamycin over the course of 14 days [191, 192]. 

Application of anti-apoptotic agents raises several concerns. The protective results of anti-apoptotic drugs are mainly based on acute studies. Therefore, the sustainability of therapeutic potential and safety remain to be evaluated in chronic exposure scenarios. There is evidence that the protective effects of caspase inhibitors to the inner ear are short term [167]. Considering that AGs are not metabolized [7, 34, 73] and remain in the hair cells for months [88, 194], potential sustainable regimens would conceivably require long-term treatment. Unfortunately, long-term treatment with anti-apoptotic drugs bears a potential carcinogenic risk, as apoptosis has a crucial primary function in preventing uncontrolled cell proliferation [195]. This carcinogenic risk, therefore, prohibits potential application in human otologic patients. Whether this risk is decreased over a long period by local application to the inner ear remains to be studied. The therapeutic application of anti-apoptotic agents to rescue hair cells after AG exposure has not been reported, but is of further translational interest.

### 4.2. Neutralization of Reactive Oxygen Species

Aminoglycosides form complexes with iron, thereby, catalyzing the formation of ROS [122]. Competitive blocking of the Fenton reaction involved by iron chelators, thus, is a reasonable approach to avoid oxidative damage from the beginning. Therefore, much efforts aiming at prevention of AG-induced hair cell death have focused on iron. Administration of the iron chelators deferoxamine and 2,3-dihydroxybenzoate before AG exposure significantly attenuated hearing threshold shifts and protected from hair cell loss *in vivo* [196–198]. 

Acetylsalicylate (ASA) is another iron chelator with additional direct antioxidant properties. ASA prevents cleavage of PKC zeta, a key regulator of NF*κβ* activated by exposure to amikacin [199]. Systemic administration of ASA effectively protects guinea pigs from gentamicin-induced hearing loss [200]. As ASA is a long-approved and routinely prescribed drug, application in human patients is the logical next step. In randomized, double-blind placebo-controlled studies, ASA significantly protected human patients from ototoxic damage without compromising the antimicrobial efficacy of gentamicin [201–203]. However, ASA itself is ototoxic and potentially causes tinnitus, vertigo, and hearing loss [204]. Although these symptoms are known to be reversible [204], AGs remain in hair cells for months [88, 194] and ototoxic damage can occur after many years [81]. Thus chronic treatment with ASA appears necessary and ototoxic effects of both, AGs and ASA, need to be evaluated over a long period. In this context, recent studies discovered a decrease of activity in auditory neurons in long-term treatment [205]. Of further concern is that AGs are frequently prescribed in children and neonates. ASA, however, is strictly contraindicated in children as it is associated with Reye's syndrome, which is a serious and often fatal disease predominantly affecting the brain and liver [206–208].

N-Acetylcysteine (NAC) is another drug commonly used in patients. Beside its mucolytic effect, NAC is also a known antioxidant. In short-term cultures of guinea pig cochlea, AG alone caused less than 30% of basal OHC survival but 90% of the apical OHC survived. This observation correlated with lower levels of the intrinsic antioxidant glutathione in basal OHC. However, survival of basal OHC was significantly improved by cotreatment with NAC as well as glutathione and salicylate [209]. In hemodialysis patients who received gentamicin treatment for bacteremia, application of NAC resulted in significantly less high frequency hearing threshold shifts compared to a control group receiving gentamicin alone. Treatment with NAC was continued for one week after cessation of the gentamicin therapy and the protective effects persisted after another six weeks [210]. Compared to ASA, NAC does not demonstrate intrinsic ototoxic side effects. 

A myriad of other agents with known antioxidant capacity has been tested for protection and treatment of AG ototoxicity. These agents are primarily antioxidants such as D-Methionine (D-Met) [211–213] and *α*-lipoic acid (*α*-LA) [214], vitamins such as *α*-tocopherol (vitamin E) [215–217] and vitamin C [218] as well as the herbal extracts Gingko biloba [219] and Danshen [220]. The hormone melatonin, normally excreted by the pineal gland, also has antioxidant capacity and successfully protected from AG ototoxicity [175, 221–223]. An alternative protective strategy against AG ototoxicity is the upregulation of intrinsic antioxidant mechanisms such as the superoxide dismutase (SOD) [209, 224, 225] (Table 2). 

Overall, antioxidants attenuate ototoxic damage from AGs. However, the majority of antioxidants did not demonstrate complete protection from AG ototoxicity [211–213, 215–217, 227, 229] and effects of long-term treatment remain to be studied.

### 4.3. Alternative Otoprotective Strategies

There exists a number of alternative approaches to protect against AG ototoxicity. One intriguing approach is moderate exposure to ototoxic stimuli with the intent to increase intrinsic antioxidant mechanisms within the ear. Exposure to low doses of amikacin or gentamicin for 30 days and consecutive high-dose treatment for another 10 to 12 days resulted in significantly less morphologic and functional hair cell damage [230, 231] (Table 3). However, this bears the undesirable risk of increased bacterial resistance and, thereby, undermines the primary antimicrobial purpose of the AG application. Exposure to moderate noise also protects from gentamicin ototoxicity in gerbils [232] (Table 3). As this does not allow for immediate application of AG in therapeutic doses, applicability in human patients appears difficult. 

Other studies successfully target NMDA receptors to protect auditory nerves [233, 234]. However, the NMDA receptor antagonists dizocilpine and ifenprodil exist as maleate and tartrate salts, which carry intrinsic metal chelating properties [235]. Their vehicle, dimethyl sulfoxide (DMSO), can also act as a radical scavenger [236]. Therefore, the results of Basile and coworkers [233, 234] were challenged by Sha and Schacht [237]. Nonetheless, NMDA antagonists do interact with receptors of afferent auditory nerve fibers [238]. Thus, targeting the auditory nerve appears reasonable as AGs interact with certain nerve synapses. AGs can aggravate myasthenia gravis and cause postoperative respiratory suppression suggesting a direct neuromuscular blockade [239–242] (Table 3). Presynaptically, AGs interfere with the calcium internalization essential for acetylcholine release [243]. At the postsynaptic level, streptomycin directly blocks the acetylcholine receptor primarily, whereas neomycin affects the open probability of the ion channel of the acetylcholine receptor [244]. Also, in rat and mouse cochlear cultures, fluorescently tagged gentamicin accumulates in the afferent auditory nerve fibers in addition to the hair cells [245].

This direct interaction with the auditory nerve also might explain therapeutic effects by neurotrophic growth factors. Ciliary neurotrophic factor (CDNF), glial-cell-line-derived neurotrophic factor (GDNF), brain-derived neurotrophic factor (BDNF), and neurotrophin 3 (NT-3) demonstrated partial protective effects against AG ototoxicity [213, 246–250] (Table 3). The contribution of neurotrophic growth factors in preventing AG ototoxicity suggests an involvement of the auditory nerve. However, there is evidence that the effects of neurotrophic growth factors are short term. Local application of BDNF (62.5 *μ*g/mL, 0.25 *μ*L/h over 28 d) to guinea pigs exposed to kanamycin (400 mg/kg, single dose, s.c.) and furosemide (100 mg/kg, single dose, i.v.) demonstrated initial protection from ototoxicity. Cessation of the therapy, however, resulted in an accelerated neuronal degeneration and after another 14 d, the survival of BDNF-treated auditory neurons did not differ from the deafened, untreated control animals [251]. 

Ethacrynic acid (EA) is a diuretic which increases AG ototoxicity when administered simultaneously [252]. Delayed co-treatment with application of EA 12–18 hr after gentamicin injections in guinea pigs resulted in significantly protected hair cell function and morphology [253]. The authors suggest that EA disrupts the blood-labyrinth barrier, thus creating a gradient promoting efflux of AG from the inner ear fluids back into the bloodstream. However, the protective effects are time dependent and could not be found when EA was injected 20 hr after the AG [253]. Moreover, simultaneous AG and EA in patients resulted in ototoxic damage after a single treatment [254], thereby excluding EA as a treatment option.

Overall, prevention of apoptotic hair cell death following AG exposure has been targeted effectively on various levels. Direct inhibition of apoptotic cascades resulted in functional and morphological preservation of hair cells. Neutralization of free radicals by antioxidants prevented activation of apoptotic enzymes. Furthermore, application of NMDA-receptor antagonists, neurotrophic growth factors, and sound conditioning have prevented ototoxic hair cell damage from AG. However, these protective results are mainly based on acute studies and the sustainability of therapeutic potential and safety remains to be evaluated in chronic exposure scenarios or in clinical trials. 

## 5. Potential Targets for Hair Cell Protection

In light of recent insight and increasing understanding of the mechanisms involved in AG ototoxicity, newer and more effective targets may be revealed in the near future. Those target sites involve the mitochondrial rRNA as well as AG entry into the inner ear fluids and hair cells. Considering the one-way valve function of the MET channel as a site of AG entry into hair cells [92, 94], the prolonged persistence of AG in hair cells poses another obstacle to overcome [194]. Therefore, avoiding entry of AG into hair cells is potentially promising. On the level of the MET channel, at least two possibilities of preventing AG entry exist. The first one involves a reversible block of the MET channel. The process of hearing requires depolarization of the inner hair cell through the MET channel [101, 260, 261]. Blocking of the MET channel would then prevent hair cell depolarization and, therefore pause hearing function. Thus, the MET channel block has to be temporary. MET channel blockers have been tested successfully *in vitro* [104]. Yet their *in vivo* effects are largely unknown. The second possibility of preventing AG entry through the MET channel involves steric modification of the chemical structure of AGs. From electrophysiological measurements, the narrowest part of the MET channel pore has been estimated to be 1.25 nm [104]. As dihydrostreptomycin is capable of blocking the MET channel [92], the difference in the dimensions of the MET channel and certain AGs appears to be small. Therefore, widening of the AG diameter by binding of inert molecules on sites irrelevant for antimicrobial activity appears a promising strategy to prohibit passage of AGs through the MET channel into the hair cells. As the passage through the bacterial membrane is self-promoting and depends on the relative positive charge of the AG [42–47, 262–264], the intended increase of size should not affect bacterial uptake of the AG as long as the polarity and the charge of the new AG molecule remains the same. However, interference with the antimicrobial activity due to sterical impairment of binding to the bacterial ribosome needs to be tested.

Another target lies in preventing AG from entering the inner ear fluids. AGs enter the inner ear fluids through the stria vascularis [87]. Blocking the passage of AG requires the identification of the transport mechanism in the blood-labyrinth barrier. 

AGs are potent antibiotics with limited application due to their side effects. Until the problem of AG ototoxicity is solved, it is crucial to be judicious in prescribing AGs for defined clinical indications. Furthermore, it is important for clinicians to remember the genetic mutations as a cause for increased susceptibility to ototoxic damage. However, indiscriminate genetic screening is not cost-effective at present. Instead, a thorough history of the patient and their family regarding ototoxic symptoms from antibiotics helps assessing the individual risk. Independent from genetic mutations, patients should undergo a baseline hearing test including ultrahigh frequencies prior to AG administration to allow for early and unambiguous assessment of potential ototoxic damage.

## Figures and Tables

**Table 1:** Overview of studies performed to protect from aminoglycoside ototoxicity by inhibition of apoptosis. C: Chicken, G: Gerbil, GP: Guinea Pig, M: Mouse, R: Rat, ZF: Zebrafish; X: *in vitro*, Y: *in vivo*; i.m.: intramuscular, i.p.: intraperitoneal, i.t.: intratympanic, s.c.: subcutaneous; d: day, h: hour; O: no effect, P/PP/PPP: partial, not significant/partial and statistically significant/complete and statistically significant protection.

Therapy (dose)	Aminoglycoside (dose)	Species	Outcome	Reference
zVAD (100 *μ*M, 26 h)	Gentamicin (0.1, 0.5, 2 mM; 24 h)	C, X	PPP	[167]
zVAD (100 *μ*M, 26 h)			PPP	
BAF (100 *μ*M, 26 h)	Gentamicin (1 mM, 6 & 24 h)	GP +	PPP	[178]
Deferoxamine (1 mM, 26 h)		G, X	PPP	
zVAD (300 *μ*M, 12 h)	Neomycin (10 *μ*M, 3 h)	ZF	PP	[179]
zVAD (local 50, 100 *μ*M or 1.5 mg/kg i.m.)	Streptomycin (1.2 g/kg; 5 d, i.m.)	C, Y	local PP i.m. P	[180]
zVAD (250 *μ*M, local @ 0.5 *μ*L/h, 14 d) zLEHD (150 *μ*M, local @ 0.5 *μ*L/h, 14 d)	Gentamicin (12 mg/mL, local @ 0.5 *μ*L/h, 14 d)	GP, Y	PP PP	[182]
zDEVD (10 or 200 *μ*M, 48 h)			PP (200 *μ*M)	
zIETD (10 or 200 *μ*M, 48 h)	Gentamicin (35 *μ*M, 48 h)	R, X	O	[193]
zLEHD (10 or 200 *μ*M, 48 h)			O	
zVAD (100 *μ*M, 26 h)		M, X	PPP	
zIETD (100 *μ*M, 26 h)	Neomycin (1 mM, 24 h)		O	[166]
zLEHD (100 *μ*M, 26 h)			PP	
d-JNKI-1 (M 2 *μ*M; GP 10 *μ*M, 1 *μ*L/h local, 7 d)	Neomycin (M 1 mM, 24–48 h; GP 300 mg/kg i.p. 5 d)	M, X GP, Y	PPP PPP	[161]
CEP-11004 (0.5 *μ*M, 84 h)	Gentamicin (50 *μ*M, 72 h)	R, X	PP	[184]
CEP-11004 (0.2, 0.4, 1.6, 4.8 *μ*M; 7 h)	Neomycin (1 mM, 3 h)	C, X	PP (1.6 *μ*M)	[152]
CEP-11004 (1.0 *μ*M; 24 h)	Neomycin (1 mM, 24 h)	M, X	PP	[183]
Estradiol (1, 10, 100, 1000 nM; 60 h)	Gentamicin (100 *μ*M, 48 h)	R, X	P	[185]
CEP-1347 (1 mg/kg s.c., 1 x/d, 14 d)	Gentamicin (120 mg/kg s.c., 1 x/d, 14 d)	GP, Y	PP	[186]
Bcl-2, transgenic	Neomycin (1 mM, 24 h)	M,X	PP	[155]
Bcl-2, transgenic	Gentamicin (40 *μ*g i.t., single dose)	M, Y	PP	[187]
Bcl-X(L), transgenic	Kanamycin (800 mg/kg, 2 x/d, s.c., 15 d)	M, Y	PP	[188]
HSP 70, transgenic	Neomycin (1–4 mM, 24 h)	M, X	PP	[191]
HSP-70, transgenic	Kanamycin (700 mg/kg, 2 x/d, s.c., 14 d)	M, Y	PP	[192]
HSP-70, transgenic	Kanamycin (700 mg/kg, 2 x/d, s.c., 14 d)	M, Y	PP	[192]

**Table 2:** Overview of studies performed to protect from aminoglycoside ototoxicity by antioxidants. C: Chicken, CH: Chinchilla, G: Gerbil, GP: Guinea Pig, H: Human, M: Mouse, R: Rat, ZF: Zebrafish; X: *in vitro*, Y: *in vivo*; i.m.: intramuscular, i.v.: intravenous, i.p.: intraperitoneal, i.t.: intratympanic, s.c.: subcutaneous, RWM: round window membrane; d: day, h: hour; O: no effect; P/PP/PPP: partial, not significant/partial and statistically significant/complete & statistically significant protection; T/TT/TTT: partial, not significant/partial and statistically significant/complete & statistically significant rescue.

Therapy (dose)	Aminoglycoside (dose)	Species	Outcome	Reference
Dihydroxybenzoate (100 mg/kg, 1 or 2 x/d, i.p., 21 or 26 d)	Gentamicin (120 mg/kg, 1 x/d, s.c., 19 d or 135 mg/kg, 1 x/d, s.c., 14 d)	GP	PP	[196]
Deferoxamine (100 mg/kg, 2 x/d, s.c., 28 d) Dihydroxybenzoate (100 mg/kg, 1 x/d, p.o.)	Gentamicin (120 mg/kg, 1 x/d, s.c., 19 d)	GP	PP PP	[197]
Dihydroxybenzoate (300 mg/kg, 2 x/d, 14-15 d)	Kanamycin (400–900 mg/kg, 2 x/d, s.c., 15 d)	M, Y	PP	[198]
Aspirin (0.1 or 1.0 mg/mL in drinking water, 8 d)	Amikacin (500 mg/kg, 1 x/d, i.p., 5 d)	R, Y	PP	[199]
Aspirin (3 × 500 mg/d, p.o., 7 d)	Gentamicin (3 × 80 mg/d, i.v., 7 d)	H	PP	[201]
Aspirin (3 × 1 g/d, p.o., 14 d)	Gentamicin (total 975–986 mg i.v./patient)	H	PPP	[202]
Aspirin (3 × 1 g/d, p.o., 14 d)	Gentamicin (80–160 mg, 2 x/d, i.v., 5–7 d)	H	PP	[203]
NAC (600 mg, 2 x/d, p.o., 22–25 d)	Gentamicin (2 mg/kg/d, i.v., avg. 15 d)	H	PP	[210]
D-Met (300 mg/kg/d, i.p., 28 d)	Amikacin (200 mg/kg, 1 x/d, 28 d)	GP, Y	PP	[211]
D-Met (200 mg/kg, 1-2 x/d, s.c., 19 d)	Gentamicin (120 mg/kg, 1 x/d, s.c., 19 d)	GP, Y	PP	[212]
L-NAME (100 *μ*M, 4 h)			PP	[213]
D-Met (50 mM, 4 h)	Gentamicin (2 mg/mL, 4 h)	GP, X	PP	
Leupeptin (1 mM, 4 h)			PP	
*α*-LA (100 mg/kg/d, i.m., 15 d)	Amikacin (400 mg/kg, 1 x/d, i.m., 15 d)	GP, Y	PP	[214]
Edaravone (3 mg/kg/d, i.p., 2–14 d)	Tobramycin (160 mg/kg, 1 x/d, s.c., 14 d)	R, Y	PPP, TTT	[226]
Resveratrol (10 *μ*M or 100 *μ*M, 24 h)	Gentamicin (0.4 mM, 24 h)	R, X	PP	[227]
*α*-Tocopherol (100 mg/kg, 1 x/d, i.m., 14 d)	Gentamicin (100 mg/kg, 1 x/d, i.m. <14 d)	GP, Y	PP	[216]
*α*-Tocopherol (100 mg/kg, 1 x/d, i.m., 14 d)	Gentamicin (100 mg/kg, 1 x/d, i.m. <14 d)	GP, Y	PP	[215]
*α*-Tocopherol (100 mg/kg, 1 x/d, i.m., 14 d)	Gentamicin (100 mg/kg, 1 x/d, i.m. <14 d)	GP, Y	PP	[217]
Glutathione (10 mM, 1 h)			PP	
Dithioerythritol (10 mM, 1 h)			PP	
Vitamin C (10 mM, 1 h)	Gentamicin (1 mM, 1 h)	GP, X	PP	[218]
Trolox (4 mM, 1 h)			PP	
Phenylene Diamine (10 mM, 1 h)			PP	
Glutathione (0.6 mL 0.3 M, p.o., 14 d)	Gentamicin (100 mg/kg, 1 x/d, i.m., 14 d)	GP, Y	PP	[228]
Ginkgo biloba (10 mg/kg, 30 min to RWM or 1 × 100 mg/kg i.p.)	Gentamicin (5 mg/kg, 45 min to RWM or 5 mg/kg, 24 h to RWM)	GP, Y	P	[219]
Danshen (1–20 mg/kg, 2 x/d, s.c., 15 d)	Kanamycin (700 mg/kg, 2 x/d, s.c., 15 d)	M, Y	PP	[220]
Melatonin (10 mg/L in drinking water p.o., 12 d or 250 *μ*g, s.c., 1 x/d, 5–12 d)	Gentamicin (160 mg/kg, 1 x/d, i.m., 5 d) Tobramycin (200 mg/kg, 1 x/d, i.m., 5 d)	R, Y	PP	[223]
Melatonin (0.4 or 4.0 mg/kg, 1 x/d, i.p., 14 d)	Amikacin (600 mg/kg, 1 x/d, i.m., 14 d)	R, Y	P (0,4 mg)	[221]
Melatonin (0.3 l/kg, 1 x/d, i.m., 17 d)	Gentamicin (120 mg/kg, 1 x/d, i.m., 17 d)	GP, X + Y	PP	[222]
Melatonin (10, 50, 100 *μ*M, 1–7 d)	Gentamicin (1 mM, 48 h)	R, X	PP	[175]
M40403 (30 *μ*M, 24 h)	Gentamicin (0.5 or 1 mM, 24 h)	M, X	PP	[225]
Cu/Zn SOD, transgenic	Kanamycin (400 mg/kg, s.c., 10 d)	M, Y	PP	[209]
Cu/Zn SOD, transgenic Mn SOD, transgenic	Kanamycin (250 mg/kg, s.c., single dose) Ethacrynic Acid (40 mg/kg, i.v., single dose)	GP, Y	O (Cu/Zn) PP (Mn)	[224]

**Table 3:** Overview of studies with alternative strategies to protect from aminoglycoside ototoxicity. C: Chicken, CH: Chinchilla, G: Gerbil, GP: Guinea Pig, M: Mouse, R: Rat, X: *in vitro*, Y: *in vivo*; i.m.: intramuscular, i.v.: intravenous, i.p.: intraperitoneal, i.t.: intratympanic, s.c.: subcutaneous; d: day, h: hour, q12 h: every 12 h; O: no effect; P/PP/PPP: partial, not significant/partial and statistically significant/complete and statistically significant protection.

Therapy (dose)	Aminoglycoside (dose)	Species	Outcome	Reference
BDNF (10 ng/mL, 4 h)	Gentamicin (2 mg/mL, 4 h)	GP, X	PP	[213]
Dizocilpine (1 mg/kg/d, osmotic pump, 14 d) Ifenprodil (10 mg/kg/d, osmotic pump, 14 d)	Neomycin (50 mg/kg, 1 x/d, s.c., 14 d) or Kanamycin (250 mg/kg, 1 x/d, s.c., 21 d)	GP, Y	PP PP	[233]
Dizocilpine (1 mg/kg, 1 x/d, s.c.,10 d)	Streptomycin (400 mg/kg, 1 x/d, s.c., 10 d)	R, Y	PP	[234]
CTNF (0.44 g/kg, 1 x/d, s.c., 30 d)	Gentamicin (80 mg/kg, 1 x/d, i.m., 30 d)	GP, Y	PP	[249]
BDNF (1 *μ*g, pellet in semicircular canal, with AG or 1 week later, over 1–8 weeks)	Gentamicin (50 *μ*g, pellet in semicircular canal, over 1–8 weeks)	CH, Y	P TT	[246]
BDNF (100 *μ*g/mL, local, pump @ 0.25 *μ*L/h, 30 d) NT-3 (100 *μ*g/mL, local, pump @ 0.25 *μ*L/h, 30 d)	Kanamycin (400 mg/kg, 1 x/d, i.p., 5 d)	GP, Y	O (BDNF) PP (NT-3)	[248]
L-NAME (100 *μ*M, 8 h) BDNF (10 ng/mL, 8 h)	Gentamicin (2 mg/mL, 8 h)	GP, X	PP PP	[247]
Isosorbide (1 mM, 8 h)	Gentamicin (2 mg/mL, 8 h)	GP, X	PP	[255]
MK 801 (1 mg/kg, 3 x before pump implantation) +/− NT-3 (local in pump @ 300 ng/h over 14 d)	Amikacin (300 mM, local pump @ 5 *μ*L/h, 24 h)	GP, Y	P (MK 801) PP (MK + NT)	[256]
GDNF (10 *μ*M, 72–96 h)	Neomycin (0.6 mM, 72 h)	R, X	P (X)	[250]
GDNF (50 ng/mL, local pump @ 0.5 *μ*L/h or single dose 0.1 mg, i.t.)	Kanamycin (200 mg/kg, s.c., single dose) + Ethacrynic Acid (40 mg/kg, i.v., single dose)	GP, Y	PP (Y)	
GDNF + TGF-1, transgenic	Kanamycin (150 mg/kg, s.c., single dose) + Ethacrynic Acid (40 mg/kg, i.v., single dose)	GP, Y	PP	[257]
GDNF, transgenic	Gentamicin (8 mg, i.t., single dose)	GP, Y	PP	[258]
GDNF, transgenic	Kanamycin (200 mg/kg, s.c., single dose) + Ethacrynic Acid (40 mg/kg, i.v., single dose)	GP, Y	PP	[259]
Gentamicin (10 mg/kg, 1 x/d, i.m., 30 d)	Gentamicin (160 mg/kg, 1 x/d, i.m., 10 d)	GP, Y	PP	[230]
Amikacin (20 mg/kg, 1 x/d, i.m., 30 d)	Amikacin (400 mg/kg, 1 x/d, i.m., 10 d)	GP, Y	PP	[231]
2 Octave-band noise (81 dB SPL for 21 d)	Gentamicin (445 *μ*g, local to RWM over 14 d)	G, Y	P	[232]
Ethacrynic Acid (40 mg/kg, i.v., single dose, 12–18 h )	Gentamicin (125 mg/kg, 1–20 injections q12h, i.m.)	GP, Y	PP	[252]
